# Acute Respiratory and Influenza Viruses Circulating in Kazakhstan During 2018–2024

**DOI:** 10.3390/pathogens14050493

**Published:** 2025-05-16

**Authors:** Tatyana Glebova, Nailya Klivleyeva, Assem Baimukhametova, Galina Lukmanova, Nurbol Saktaganov, Nuray Ongarbayeva, Baiken Baimakhanova, Gulmira Kassymova, Madisha Sagatova, Almagul Rachimbayeva, Nazgul Zhanuzakova, Tatyana Naidenova, Nigina Rakhmonova, Richard Webby

**Affiliations:** 1 The Research and Production Center for Microbiology and Virology, Almaty 050010, Kazakhstan; taty1962@mail.ru (T.G.); a_baimukhametova@mail.ru (A.B.); gal_l@bk.ru (G.L.); nsaktaganov1984@mail.ru (N.S.); nuray.syrlybay@gmail.com (N.O.); bbbayken@mail.ru (B.B.); 2 Zhambyl Regional Multidisciplinary Center of Oncology and Surgery, Health Department of the Akimat of Zhambyl Region, Taraz 080003, Kazakhstan; terapiya2014@mail.ru; 3 The East Kazakhstan Regional Branch of National Center for Expertise, 17 Independence Avenue, Ust-Kamenogorsk 070003, Kazakhstan; madisha_sagatova@mail.ru; 4 The Almaty Branch of National Center for Expertise, 3 Zhibek Zholy Avenue, Almaty 050000, Kazakhstan; kalievna72@mail.ru; 5 Scientific Center of Pediatrics and Pediatric Surgery, 146 Al-Farabi Avenue, Almaty 050040, Kazakhstan; biochem_vir@mail.ru; 6 Regional Clinical Hospital of the Health Department of the Karaganda Region, 10 A Nursultan Nazarbayev Avenue, Karaganda 100000, Kazakhstan; galina.owl@gmail.com; 7 LLP “Regional Consultative and Diagnostic Medical Center Sadykhan”, 64 Tole bi Avenue, Taraz 080000, Kazakhstan; aniris_nigina@mail.ru; 8 Department of Infectious Diseases, St. Jude Children’s Research Hospital, Memphis, TN 38105-3678, USA; richard.webby@stjude.org

**Keywords:** respiratory infection, influenza, virus, antigen, diagnosis

## Abstract

Respiratory tract infections cause serious morbidity and mortality and are a major public health problem. The objective of our study was detection of the prevalence of viral respiratory diseases in the territory of Kazakhstan during the epidemic period of 2018–2024. The presence of respiratory viruses in nasopharyngeal swabs was analyzed using real-time polymerase chain reaction. The level of specific antibodies in the blood serum was determined by hemagglutination inhibition assay and enzyme-linked immunosorbent assay. In rtRT-PCR, patients were diagnosed with non-influenza viral respiratory tract infections as well as influenza viruses A(H1N1), A(H3N2), and B. Antibodies were detected against A(H1N1)pdm09, influenza A(H3N2), and influenza B viruses and with simultaneous detection of both viruses. The circulation of influenza A(H3N2) viruses belonging to the 3C.2a1b.2a.2a.3a.1 clade was confirmed by whole-genome sequencing. According to the results, in the period 2018–2024, the spread of influenza A and B viruses and non-influenza respiratory tract infections was observed. The data of this study confirm the role of known causative agents of epidemic infection and indicate the need to continue monitoring their spread in Kazakhstan, which may add to the general quality of the health system.

## 1. Introduction

Viruses play an important role in the etiology of infectious diseases. The incidence of respiratory viral infections (ARVI), including influenza, remains high and increases annually in the autumn–winter period. This is due to the easier transmission of infection, which is enabled by crowded premises. The rate of upper respiratory tract infections was found to be 20–40% of outpatient hospitalizations and 12–35% of inpatient hospitalizations [1,2]. Currently, the role of non-influenza respiratory viruses has increased following the emergence of SARS-CoV-2 in 2019. However, influenza, along with SARS-CoV-2, represents a huge public health problem, as it causes severe morbidity and mortality [3]. Rapid reproduction and high mutation rates characteristic of influenza A viruses result in the emergence of viruses with new antigenic properties. Due to the presence of a segmented genome, simultaneous infection of a host cell with several influenza viruses of different origin (birds, humans, and other mammals) can lead to genetic reassortment [4,5,6]. The reassortment of viral genomes obtained from different strains and the constant process of mutation of influenza viruses leads to annual epidemics and sometimes pandemics due to the emergence of zoonotic strains that are highly pathogenic for humans [7].

Furthermore, the rapid variability of the influenza A virus leads to a decrease in population immunity and vaccine effectiveness. It is precisely because of the high mutation rate that influenza remains a serious threat to global public health [8,9].

Ongoing influenza surveillance plays a vital role in identifying unique influenza viruses in humans or new genetic variants of influenza viruses of epidemiological and clinical significance [10]. In addition to molecular methods, serological methods of research are still widely used in the diagnosis of influenza infection. Serological diagnostics play a role in the diagnosis of respiratory infection in cases of atypical or asymptomatic disease and in predicting the epidemic situation [11]. Influenza surveillance information is essential for the selection of representative viruses for vaccines, which is carried out every two years and determines their needs in each region of the world [12].

The objective of our study was to detect the prevalence of viral respiratory diseases in the territory of Kazakhstan during the epidemic periods of 2018–2024.

## 2. Materials and Methods

Biosamples (5499 nasopharyngeal swabs and 1521 blood serum) were received from patients of medical institutions located in various regions of the Republic of Kazakhstan (southern, eastern, northern, central, and western parts of Kazakhstan) during the epidemic seasons of 2018–2024. The study included biological samples obtained from patients hospitalized with symptoms of acute respiratory viral infection, bronchitis, pneumonia, and others. The sample was balanced by gender. For detailed analysis, the sample was divided into six age groups: 0–4, 5–9, 10–17, 18–30, 31–64, and >65 years.

For diagnosing the causative agent of acute respiratory viral infections, the collection of biomaterials was carried out in accordance with the recommendations of WHO [13]. The study was conducted according to the guidelines of the Declaration of Helsinki (1975, revised in 2013) and approved by the Institutional Review Board of the Local Bioethical Committee Limited Liability Company “Research and Production Center for Microbiology and Virology”(protocol code No. 19, 20 April 2025). The collection of biological material was carried out with the participation of the physicians and middle-level medical personnel of medical institutions.

Pharyngeal and nasal smears were collected from patients with symptoms of respiratory infections no later than the third day from the onset of the disease using sterile viscose swab probes with plastic holders. When taking a swab from the pharynx, the swab was pressed tightly to the hyperemic areas in the tonsil area and the back wall of the pharynx, carefully wiping these areas with the swab. To take a swab from the nose, the swab was inserted alternately into each nostril deep enough to reach the area of the lower nasal passage. After this, the swabs were placed in test tubes with transport medium (DMEM cell culture medium with the addition of a mixture of antibiotics gentamicin (50 μg/mL) and amphotericin B (2.5 μg/mL) and bovine serum albumin fraction V (0.5%). Nasopharyngeal swabs were transported and then stored in liquid nitrogen. Repeated freezing and thawing of specimens was avoided. Serum was separated by centrifugation at 400× *g* for 15 min and stored at −20 °C until analysis.

The collected nasopharyngeal swabs were analyzed for the presence of the viruses studied using real-time polymerase chain reaction (rtRT-PCR) with hybridization-fluorescence detection on a Rotor-Gene Q6 plex amplifier (QIAGEN, Hilden, Germany). To extract nucleic acids, the following reagent kits were used: QIAamp Viral RNA Kits for RNA Extraction (QIAGEN, Germany) and AmpliSens^®^ RIBO-prep, AmpliSens^®^ RIBO-sorb (Federal Budgetary Scientific Institution Central Research Institute of Epidemiology of Rospotrebnadzor, Moscow, Russia) according to the manufacturer’s instructions. To synthesize complementary DNA (cDNA) from RNA isolated from biological samples, the REVERTA-L reagent kit from the last manufacturer was used. Detection of the type influenza virus were performed with the AmpliSens^®^ Influenza virus A/B-FL kits. To determine the subtypes of IAV, the following kits were used in accordance with the their instructions: AmpliSens^®^ Influenza virus A/H1-swine-FL, AmpliSens^®^ Influenza virus A-type-FL, and AmpliSens^®^ Influenza virus A-type-H5, H7, and H9-FL and other respiratory viruses kits, namely AmpliSens^®^ ARVI-screen-FL and AmpliSens^®^ SARS-CoV-2-IT [14,15].

Nonspecific inhibitors in sera were removed by treatment for 18 h at 37 °C with a receptor-destructive enzyme in a working dilution of 1:50, at the rate of three volumes of a receptor-destructive enzyme per volume of serum. Subsequently, the serum was diluted to 1:10 by adding six parts of the phosphate-buffered saline solution and heated at 56 °C for 30 min. The level of specific antibodies to influenza virus hemagglutinins in blood serum was determined using HI and ELISA according to WHO recommendations [13]. HI was performed using IDS A/Michigan/45/2015 (H1N1)pdm, IDS A/Singapore/INFIMH-16-0019/2016 (H3N2), IDS B/Phuket/3073/13 (Yamagatskaya line), IDS B/Colorado/06/2017, IDS influenza B/Washington/02/2019, IDS influenza A(H1N1)pdm 09 (A/Guangdong-Maonan /SWL1536/2019), IDS influenza A(H3N2) Hong Kong 2671/2019, IDS influenza A(H1N1)pdm09, and IDS influenza A/Victoria/4897/2022 (LLC “Enterprise for the Production of Diagnostic Preparations”, Moscow, Russia). ELISA was performed using the kits ELISA TS IgG to influenza virus A(H1N1) and ELISA TS IgG to influenza virus A(H3N2) as well as ELISA TS IgG to influenza virus B from the same manufacturer.

The whole-genome amplification of influenza viral genetic material was obtained by single-step reverse transcription and polymerase chain reaction using the RT-PCR reagent kit Biomaster RT-PCR-RV (2×) (Biolabmix, RF, Novosibirsk, Russian Federation) in accordance with the modified protocols for influenza A viruses [16] on Bio-Rad CFX96 Touch devices (Moscow, Russian Federation). The results of the obtained PCR products (fragments) of the whole-genome amplification of influenza viruses were verified by electrophoresis on 2% agarose gel in Tris-acetate-EDTA buffer.

Library preparation for whole-genome sequencing was carried out using the Illumina DNA Prep kit (Illumina, San Diego, CA, USA) according to the manufacturer’s recommendations. The samples were indexed using the Illumina kit IDT^®^ for Illumina^®^ DNA/RNA UD Indexes Set A/B/C/D. The products of whole-genome amplification of the influenza virus were purified using AMPure XP magnetic beads (Beckman, Indianapolis, IN, USA). The concentration of double-stranded DNA in PCR products was determined using a Qubit fluorometer and the Qubit HS dsDNA kit (Life Technologies, Carlsbad, CA, USA). The purified cDNA was diluted to a concentration of 0.2 ng/µL, after which it was used to prepare the library with the Illumina reagent kit. Sequencing was performed using the Illumina NextSeq P2 100-cycle kit on the Illumina NextSeq 2000 sequencer (Illumina, USA). Evolutionary history was inferred using the maximum likelihood method based on the Hasegawa-Kishino-Yano model [17]. Initial tree for the heuristic search was obtained automatically by applying neighbor-joining and BioNJ algorithms to a matrix of pairwise distances estimated using the maximum composite likelihood (MCL) approach and then selecting the topology with the superior log likelihood value. A discrete Gamma distribution was used to model evolutionary rate differences among sites (five categories (+G, parameter = 0.1000)). The tree was generated using neighbor-joining method, tested by bootstrapping for 1000 replicates, and drawn to scale, with branch lengths measured in the number of substitutions per site. The analysis involved 54 nucleotide sequences. All positions containing gaps and missing data were eliminated. There was a total of 1697 positions in the final dataset. Evolutionary analyses were conducted in MEGA7 [18].

Statistical analysis was carried out using Microsoft Excel application packages and GraphPad Prism software version 9.1. (GraphPad Software, La Jolla, CA, USA), with Two-way ANOVA. For all data, the arithmetic means and standard deviations from the mean were calculated. The significance of differences between experimental data was determined. Differences were considered significant at *p* < 0.05. Statistical analysis of samples with normal distribution was performed using parametric criteria. For those that did not obey the law of normal distribution, the analysis was performed using nonparametric criteria [19].

## 3. Results

Nasopharyngeal swabs from 5499 individuals of different ages (born 1934–2023) with various respiratory diseases were examined to study the prevalence of ARVI pathogens during 2018–2024.

**Figure 1:**
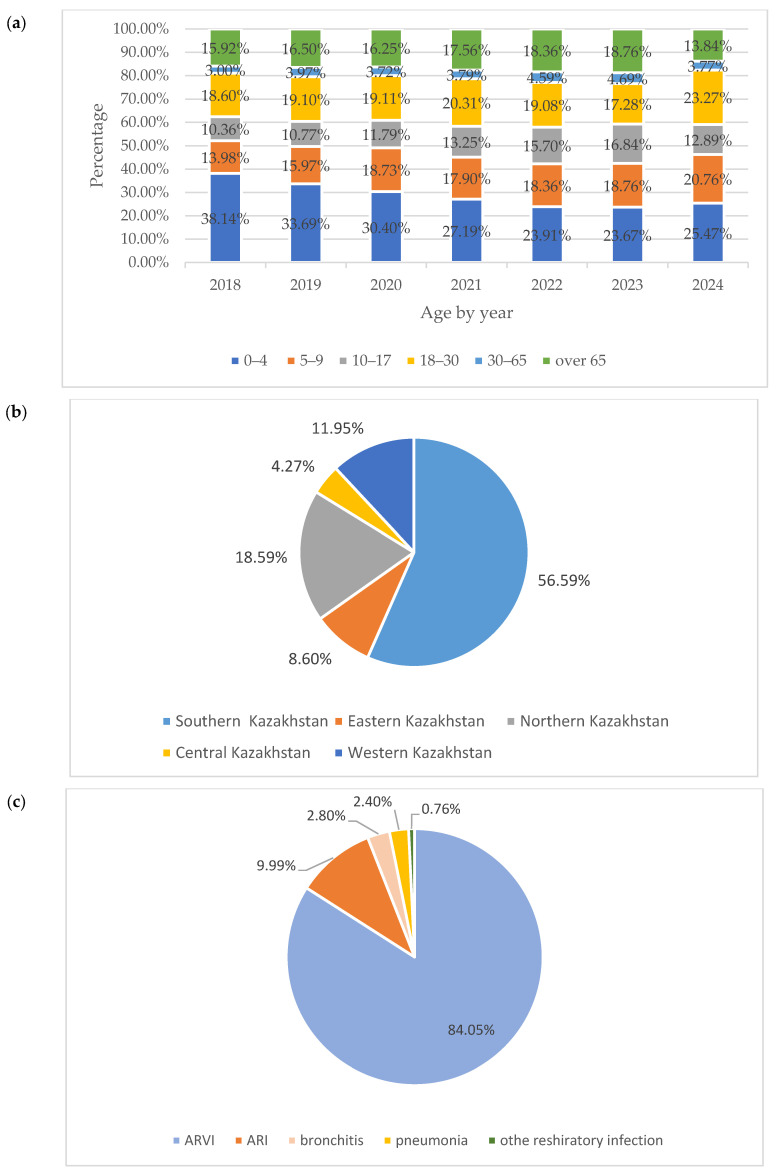
Distribution of samples according to age, sampling points, and primary diagnosis of the studied individuals. Distribution of samples according to (**a**) age, (**b**) sampling points, and (**c**) respiratory diseases of individuals studied.

Most of the samples were collected from children up to and including 17 years of age, representing 60.51% of the total sample. The second largest group was represented by individuals of working age (18–64 years) and made up 22.90% of the sample. The samples collected from people of retirement age constituted approximately 16.58% (Figure 1a).

Most of the samples were obtained from clinics located in the southern part of Kazakhstan (Almaty, Zhambyl, South Kazakhstan, and Kyzylorda regions), as large cities with high population densities are in this part of Kazakhstan (Figure 1b).

The maximum number of samples (84.05%) was obtained from patients diagnosed with acute respiratory infection. Samples from patients diagnosed with ARVI, pneumonia, and bronchitis constituted a smaller proportion (2.80% to 9.99%). Less than 1% of samples were collected from patients with other respiratory diseases (rhinitis, pharyngitis, sore throat, tonsillitis, and asthma) (Figure 1c).

As a result of rtRT-PCR testing, positive samples were detected in 2049 samples (37.26% of the total number of samples tested) (Table 1). The genetic material of non-influenza respiratory pathogens—hAdv, hBov, hCov, hMpv, hPiv types I-IV, hRSv, and hRv—was detected in 752 (13.68%) samples. The largest proportion of positive samples of respiratory infections of non-influenza etiology fell to hRSv 489 (8.89%) and hRv 142 (2.58%). A smaller proportion was noted for hAdv, hBov, hCov, hMpv, and hPiv types I and III (less than 1% of samples). However, hCov and hMpv were not detected in the circulating samples of 2021–2022. In addition, hPiv types II and IV were not detected in the samples during the entire study period since these viruses are much less common in the population [20]. Influenza virus genetic material was detected in 1297 swabs (23.59% of the total number of samples tested): influenza A virus in 1269 samples (23.08%) and influenza B virus in 28 (0.51%). Influenza A(H1N1)pdm09 virus RNA was detected in 598 samples (10.87%) and influenza A(H3N2) virus in 331 (6.02%). In 340 samples (6.18%) positive for influenza A virus, the subtype could not be determined. A(H1N1)pdm09 was the dominant agent among influenza viruses during the study period. However, in the epidemic period of 2023–2024, its detection rate was minimal and amounted to 0.31%. During this period, the main etiological agent in the structure of influenza morbidity was the A(H3N2) virus, which accounted for 19 (5.97%) samples.

In the serological examination of 1521 serum samples, the percentage of females examined was 52.07% (792 samples), while the percentage of males was slightly lower at 47.93% (729 samples). The most numerous were age groups of 30 to 65 years and the elderly (older than 65 years), whose percentages were 41.22% (627 samples) and 38.79% (590 sera), respectively. Fewer samples were obtained from children 5–9 years of age: 1.91% (29 samples) (Table 2).

**Figure 2:**
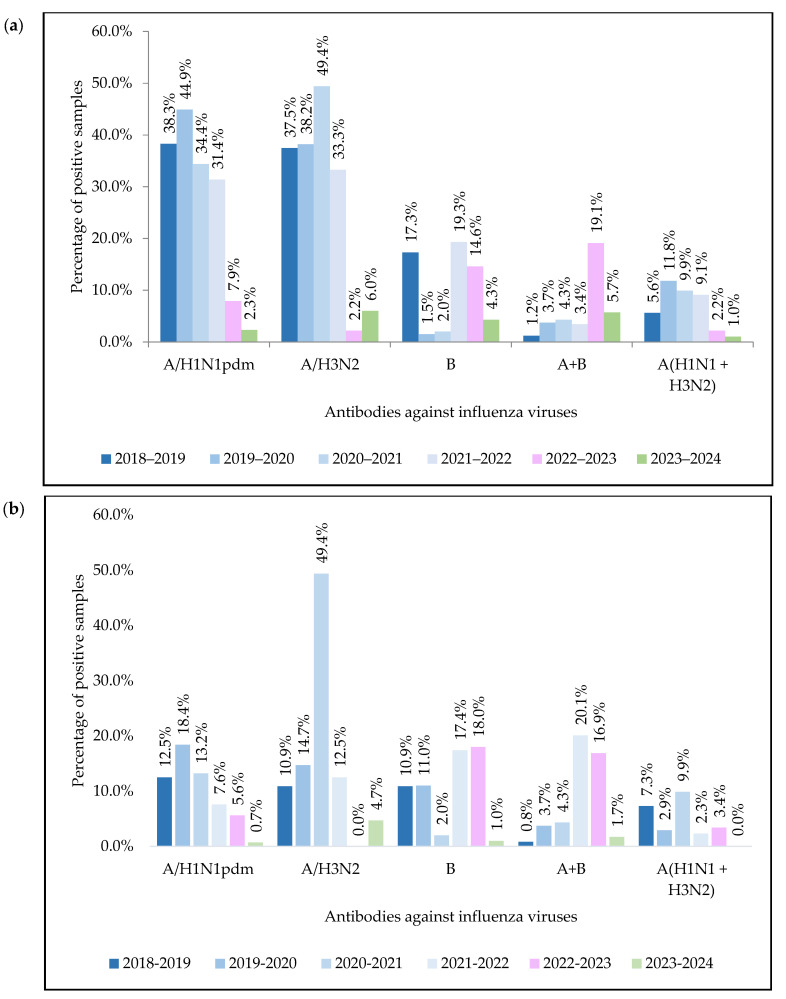
Detection of antibodies against influenza viruses in blood serums (**a**) using hemagglutination inhibition assay; (**b**) using enzyme-linked immunosorbent assay.

Serological assays using HI showed the presence of antihemagglutinins of influenza A(H1N1)pdm09 virus in 396 (26.04%) samples, influenza A(H3N2) virus in 450 (29.59%), and type B virus in 143 (9.40%). Antibodies to the two subtypes of influenza A virus and type B virus were detected simultaneously in 85 (5.59%) cases, and antibodies to both influenza A viruses (H1N1+H3N2) were detected in 100 (6.57%) (Figure 2).

In ELISA, antibodies to influenza A(H1N1)pdm virus were detected in 140 samples (9.2% of cases), to A(H3N2) virus in 152 (10.0%), and to type B virus in 146 (9.6%). Antibodies to two influenza A virus subtypes simultaneously were detected in 58 (3.8%) sera, and antibodies to influenza A and B viruses were detected in 148 (9.7%) (Figure 2). It should be noted that no antibodies to the influenza A(H3N2) virus were detected in serum specimens during the epidemic period 2022–2023.

**Figure 3:**
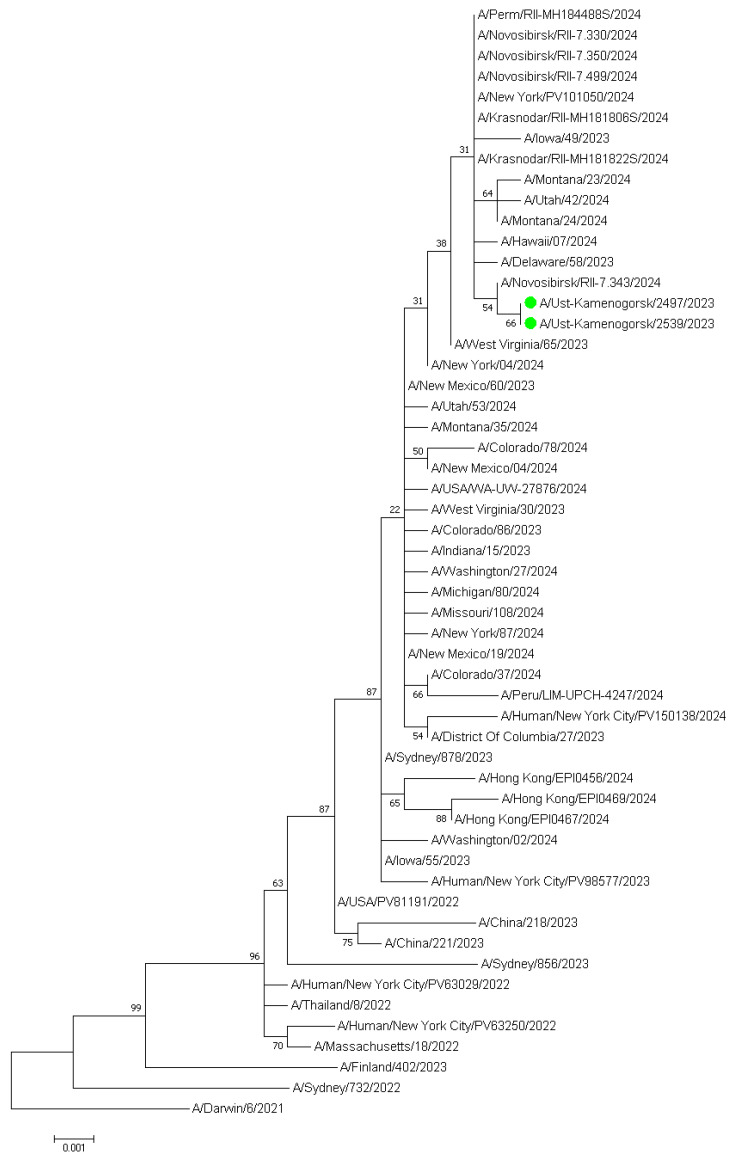
Molecular phylogenetic analysis for H3 subclade classification by maximum likelihood method. The tree with the highest log likelihood (−3146.42) is shown. The percentage of trees in which the associated taxa clustered together is shown next to the branches. The green colored circles represent the hemagglutinin nucleotide sequences of two samples from Ust-Kamenogorsk (eastern part of Kazakhstan).

Direct sequencing of 37 positive nasal samples from symptomatic patients yielded partial IAV genome sequences for eleven samples, and whole-genome assembly was performed on two samples from Ust-Kamenogorsk (eastern part of Kazakhstan). The molecular phylogenetic analysis for H3 subclade classification by maximum likelihood method of two influenza A/Ust-Kamenogorsk/2497/2023 and A/Ust-Kamenogorsk/2539/2023 (H3N2) viruses is presented in Figure 3.

The results show that the strains are more closely related to influenza A/Novosibirsk/RII-7.343/2024, A/Delaware/58/2023, A/Hawaii/07/2024, A/Krasnodar/RII-MH181822S/2024 viruses, and other strains from the United States and various regions of the Russian Federation. The strains were found to be closely related to the A/District of Columbia/27/2023, which has been recommended for use in vaccines for the 2025 Southern Hemisphere influenza season, as well as to the A/Thailand/8/2022 and A/Massachusetts/18/2022 vaccine strains, and they belong to the 3C.2a1b.2a.2a.3a.1 clade.

The sequences were registered in the GISAID database with the accession numbers EPI_ISL_19186465 and EPI_ISL_19186461, respectively. From genotyping using FluSurver analysis tools of the GISAID database, viruses were found to have positions indicating their sensitivity to NA inhibitors—zanamivir, peramivir, oseltamivir, and laninamivir—and to the PA inhibitor baloxavir on the GISAID platform: https://platform.epicov.org/epi3/cfrontend#26e134 (accessed on accessed on 13 May 2025) [21].

Thus, the results of the rtRT-PCR screening of nasopharyngeal swabs indicate the circulation of influenza type A: H1N1pdm09 and H3N2 viruses in the population in 2018–2024, with different proportions in different epidemic periods and with the variable predominance of one of them. Among non-influenza viruses, the most frequently detected were hRSv and hRv. According to HI assay and ELISA data, influenza type A and B viruses were circulating during the epidemic period 2018–2024, with influenza A viruses predominating. The obtained phylogenetic data show that the strains that circulated in Kazakhstan in 2023–2024 belong to the 3C.2a1b.2a.2a.3a.1 clade.

## 4. Discussion

Circulation of influenza pathogens and other ARVIs plays a leading role in the etiology of respiratory viral infections [22]. ARVIs can cause significant morbidity and mortality among children under 5 years of age, the elderly, people with chronic health problems (chronic diseases of the heart, lungs, kidneys, liver and blood and metabolic and neurological development disorders), as well as people with immunosuppressive conditions (HIV infections, chemotherapy or steroid treatment, and malignant neoplasms) [23]. Over the last century, the picture of influenza epidemics has changed dramatically. Simultaneously with the spread of the known influenza viruses A(H1N1), A(H3N2) and B, the influenza virus A(H1N1)pdm09 has become widespread, and the role of respiratory viruses of non-influenza etiology has increased [24,25].

An estimated 10% of all children under 18 years of age and 4% of the total population aged <65 years seek outpatient care for influenza-related respiratory illness each year [26]. In our study, the largest age group with ARVI symptoms were children (0–17 years), comprising 60.52% of the sample; the adult population accounted for 22.90%, and the elderly comprised 16.58%. In Kazakhstan, the average age of the population is 31.94 years, and the proportion of children and adolescents under 17 years old makes up 33.97% of the Kazakhstani population [27]. This may explain the high percentage of positive samples in the pediatric age group.

The emergence of SARS-CoV-2 in 2019–2020 has had a significant impact on the circulation of other respiratory viruses. During the COVID pandemic, for example, influenza and RSV viruses temporarily disappeared from the epidemic picture. In the post-pandemic period, there has been a partial change in the seasonality and epidemiology of respiratory viruses due to the influence of co-circulation of SARS-CoV-2 [28,29,30].

According to the analysis of data on various coinfections of influenza and SARS-CoV-2, it was found that the most common coinfection pathogens were influenza viruses and enteroviruses. Children were significantly more likely to have SARS-CoV-2 coinfection than adults, and critically ill patients were more likely to have coinfections. In addition, patients with respiratory co-infection have a higher risk of death [31]. Our study did not reveal cases of coinfection with influenza viruses and other respiratory viruses. Data from other researchers also indicate a low frequency of coinfection with respiratory viruses [29,32,33,34]. Despite the relatively low proportion of patients with respiratory viral co-infection and due to the high risk of its negative consequences, careful monitoring of matches and interactions between SARS-CoV-2 and other respiratory viruses using multiplex viral panel testing in patients with compatible symptoms is necessary [31,35].

Higher rates of influenza and other respiratory virus infections have been reported in various countries around the world [36,37,38,39]. So, high levels of hRSv and hRv have been detected in patients with respiratory infections in a number of countries in Europe [40,41], Asia [37,38,42,43,44,45,46,47], and Africa [48]. In a number of Asian countries, the leading role of hMPV in the non-influenza viral etiology of respiratory infections has been noted, while the emergence of new hMPV variants has also been noted [36,37,38,49,50]. In general, it is necessary to note the diverse nature of the territorial distribution and proportion of respiratory viruses depending on the age group of patients [51,52].

In our study, out of 5499 samples tested by rtRT-PCR, a positive result for influenza was obtained in 1297 samples, which amounted to 23.59%, while other non-influenza respiratory viral infections were detected in 752 samples (13.68%). Serological analysis of 1521 blood serum samples using HI and ELISA revealed the presence of antibodies to influenza A(H1N1)pdm09 virus in 26.04%/9.2% of samples, influenza A(H3N2) virus in 29.59%/10.0%, and influenza type B virus in 9.40%/9.6%. Antibodies to both influenza A viruses (H1N1+H3N2) were detected in 6.57%/3.8% of cases, and antibodies to influenza A and influenza B viruses were detected simultaneously in 5.59%/9.7% of cases. It should be noted that in the period 2022–2023, antibodies to the influenza A(H3N2) virus were not detected in sera.

Study of the etiological structure of non-influenza ARI in Kazakhstan in 2016–2019 has revealed the predominance of hRSv (14.45%) and hRv (3.69%), while hPiv, hMpv, and hAdv were detected much less frequently (about 1%). HBov and hCov were detected in isolated cases [2,53,54]. During the pandemic period and 2020–2021, there was an unprecedented decline in the circulation of the influenza virus in the world as well as in Kazakhstan. This may be due to factors such as viral interference against the background of COVID-19 infection as well as measures to mitigate the pandemic [55]. However, hRSv and hRv have not lost their dominant role among other non-influenza respiratory viruses; the share of these viruses in 2020–2022 remained within 2.86% and 1.36%, respectively. The share of hPiv and hBov accounted for up to 0.39%, while these figures for hAdv, hCov, and hMpv decreased significantly (from 0.19% to 0.03%). In general, it is necessary to note the diverse nature of the territorial distribution and proportion of respiratory viruses depending on the age group of patients [51,52]. In the post-pandemic period, the incidence of viruses causing respiratory diseases among the population of Kazakhstan is gradually increasing (37.17%); however, the 2017–2020 figures have not yet been obtained [2,53,54].

In Russia, in 2021–2022, the circulation of influenza viruses resumed with a predominance of new clusters of A(H3N2), and in 2022–2023, most viruses belonged to the A(H1N1)pdm09 subtype, representing the new antigenic clade 6B.1A.5a.2a. A smaller proportion belonged to influenza B, representing the new clade V1A.3a.2. Phylogenetic analysis of the HA gene sequences of influenza A(H3N2) viruses showed that they belong to one of the new genetic subclades of the 3C.2a1b.2a.2 clade. The dominant viruses in the 2022–2023 flu season were A(H1N1)pdm09, which reemerged after two years of almost complete absence during the COVID-19 pandemic [55]. In Kazakhstan, which has a long border with Russia, in 2022–2023, 12.29% of positive samples were also influenza A(H1N1)pdm09 viruses. Meanwhile, in the 2023–2024 epidemic season, the predominant agent among influenza viruses was the A(H3N2) virus.

In the United States, there was a surge in influenza cases in 2022–2023, likely due to a relaxation of pandemic measures, a decrease in the number of influenza vaccinations, and reduced levels of post-vaccination and natural antibodies due to a break in influenza circulation in 2020–2021 as well as an antigenic shift. After the COVID-19 pandemic, influenza contagiousness increased due to a relaxation of pandemic protection measures [56,57,58,59].

Retrospective analyses also play an important role in epidemiological surveillance systems. Serological methods are the oldest methods used in the diagnosis of viral diseases, and are still widely used in clinical trials due to their versatility, specificity, ease of use, and low cost. Antiviral immunoglobulins and viral diagnostic antigens allow the use of serological methods in the diagnosis and monitoring of infectious diseases and groups of infections. HI and ELISA tests are most widely used to detect specific antibodies in blood serum to detect population immunity [60].

**Figure 4:**
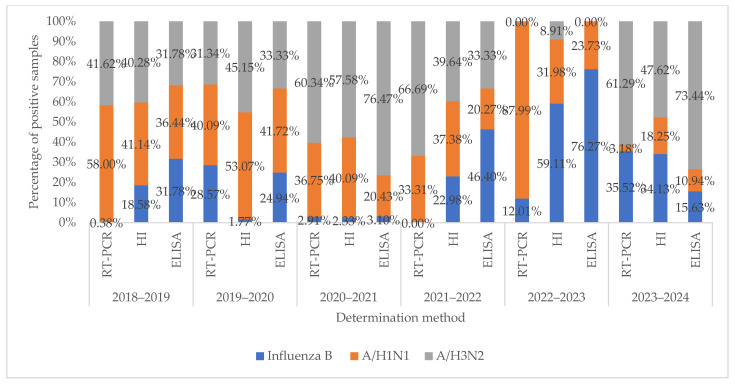
Correlation between rtRT-PCR data and serological methods for studying the circulation of influenza viruses.

When comparing the data obtained by rtRT-PCR on the prevalence of influenza viruses in nasopharyngeal swabs from 2018 to 2024 and the presence of antibodies in HI and ELISA during this period, a correlation was found between these data (Figure 4). Detection of antibodies to the influenza virus in serological studies is mainly associated with information on the spread of the influenza virus during a certain period of the epidemic. However, in contrast to the rtRT-PCR data showing a low frequency of detection of the influenza B virus in 2018–2019 and 2021–2022, the presence of antibodies to this influenza virus was detected more often in serological studies. Such a discrepancy may indicate the persistence of antibodies to the influenza virus after the previous epidemic or induced immunity after vaccination.

The use of serological tests in this work, such as HI and ELISA, revealed that they can be an additional tool in examining patients with ARVI to detect specific antibodies in the blood. Detection of antibodies is indirect evidence of the influenza virus role in the occurrence of ARVI and their interaction with the human body. Serological diagnostics is especially important in the atypical or asymptomatic course of influenza-like infections to determine the etiology of ARVI by detecting specific antibodies and confirming prognoses.

Susceptibility to influenza may be affected by factors such as immune status, pregnancy, gender, concomitant diseases, and age. A high proportion of antibodies to influenza A(H1N1)pdm viruses was found in individuals aged 10–17 years, while the highest proportion of antibodies to influenza A(H3N2) viruses was found in individuals aged 30–65 years; after 65 years, the presence of antibodies to influenza viruses decreased.

We analyzed data obtained during the monitoring of acute respiratory viral infections and influenza according to standardized international protocols. Unfortunately, this has several limitations. The first is the small sample size relative to the population of Kazakhstan. Secondly, the collection of samples from all regions was not evenly represented, which distorts the comparison of the data. Despite the fact that biological samples were obtained in various regions of the Republic of Kazakhstan, most of them were obtained from large cities with a high population density, located in the southern part of Kazakhstan. Furthermore, the period we analyzed (2018–2024) includes the COVID-19 pandemic, which has had a significant impact on the decrease in the detection of ARVI and influenza circulation due to the introduction of restrictive measures, including quarantine (strict isolation regime) as well as reduced patient access to medical services. However, the detection of influenza viruses A(H1N1)pdm, A(H3N2), and B confirms their role in the epidemic process and indicates the need to continue monitoring the epidemiological spread of respiratory viruses in various regions of Kazakhstan. Due to the inherent genetic variability of influenza A viruses (antigenic drift and antigenic shift), new virus variants appear with new biological and antigenic properties, resulting in annual outbreaks and epidemics of influenza. To confirm influenza forecasts and exclude other diseases caused by various viral and bacterial agents, ongoing laboratory studies with detailed antigenic and genetic analysis of seasonal influenza viruses are required. Ongoing research in this area is vital to identify representative strains and determine both the components of seasonal influenza vaccination and the vaccination period to prevent large outbreaks and epidemics [61].

## 5. Conclusions

Comprehensive study of respiratory virus circulation in Kazakhstan in 2018–2024 revealed the leading role of influenza viruses A(H1N1)pdm09, A(H3N2), and B, which were detected in different epidemic seasons with different intensities, with the variable predominance of one of them. The phylogenetic data obtained show that the strains circulating in Kazakhstan in 2023–2024 belong to the 3C.2a1b.2a.2a.3a.1 clade. Among non-influenza viruses, hRSv and hRv were detected most frequently. Susceptibility to influenza is influenced by factors such as immune status, pregnancy, gender, concomitant diseases, and age. The data obtained emphasize the need for continuous monitoring of the spread of respiratory viruses in both Kazakhstan and the world. Studying the spread of respiratory viruses in Kazakhstan will help in identifying representative strains suitable as components of seasonal vaccines and selecting vaccination regimens against respiratory infections.

## Figures and Tables

**Table 1:** Screening of nasopharyngeal samples collected in Kazakhstan in 2018–2024 using rtRT-PCR.

Virus	Number of PCR-Positive Virus Samples by Year								
	2017–2018	2018–2019	2019–2020	2020–2021	2021–2022	2022–2023	2023–2024	Total	*p*-Value
Number of tested samples	1602	1309	806	581	414	469	318	5499	0.0591
Adenovirus (hAdv)	14 (0.87) ^1^	13 (0.99)	1 (0.12)	1 (0.17)	1 (0.24)	2 (0.43)	1 (0.31)	33 (0.60)	0.0809
Bocavirus (hBov)	2 (0.12)	1 (0.08)	2 (0.25)	2 (0.34)	2 (0.48)	1 (0.21)	1 (0.31)	11 (0.20)	0.0543
Coronavirus (hCov)	10 (0.62)	8 (0.61)	4 (0.50)	0	0	2 (0.43)	1 (0.31)	25 (0.45)	0.0742
Metapneumovirus (hMpv)	11 (0.69)	10 (0.76)	1 (0.12)	0	0	1 (0.21)	0	23 (0.42)	0.0923
Parainfluenza (hPiv) 1/3	10 (0.62)	9 (0.69)	2 (0.25)	2 (0.34)	2 (0.48)	2 (0.43)	2 (0.63)	29 (0.53)	0.0660
Parainfluenza (hPiv) 2/4	0	0	0	0	0	0	0	0	0
Respiratory syncytial virus (hRSv)	201 (12.55)	214 (16.35)	22 (2.73)	14 (2.41)	13 (3.14)	15 (3.20)	10 (3.14)	489 (8.89)	0.0842
Rhinovirus (hRv)	68 (4.24)	41 (3.13)	7 (0.87)	7 (1.20)	7 (1.69)	8 (1.71)	4 (1.26)	142 (2.58)	0.0782
ARVI in total	316 (19.73)	296 (22.61)	39 (4.84)	26 (4.48)	25 (6.04)	31 (6.61)	19 (5.97)	752 (13.68)	0.0804
Influenza A non-detected	216 (13.48)	15 (1.15)	13 (1.61)	0	0	70 (14.93)	26 (8.18)	340 (6.18)	0.0885
Influenza B	0 (0.00)	2 (0.15)	10 (1.24)	2 (0.34)	0	3 (0.64)	11 (3.46)	28 (0.51)	0.0753
A(H1N1)pdm09	213 (13.30)	301 (22.99)	14 (1.74)	25 (4.30)	22 (5.31)	22 (4.69)	1 (0.31)	598 (10.87)	0.0871
A(H3N2)	0 (0.00)	216 (16.50)	11 (1.36)	41 (7.06)	44 (10.63)	0	19 (5.97)	331 (6.02)	0.0983
Influenza in total	429 (26.78)	534 (40.79)	48 (5.96)	68 (11.70)	66 (15.94)	95 (20.26)	57 (17.92)	1297 (23.59)	0.0738
A total of positive samples	745 (46.50)	830 (63.41)	87 (10.79)	94 (16.18)	91 (21.98)	126 (26.87)	76 (23.90)	2049 (37.26)	0.0729

^1^ Data represent the number of positive samples (percentage).

**Table 2:** Detection of antibodies against influenza virus strains in different age groups using HI assay and ELISA during the 2018–2021 epidemic period.

Age Group (Years)	Sample Size	Numbers of Participants That Are Immune to Influenza Viruses							
		A(H1N1)pdm		A(H3N2)		Type B		Total	
		HI	ELISA	HI	ELISA	HI	ELISA	HI	ELISA
0–4	64 (4.21) ^1^	17 (26.56)	11 (17.19)	33 (51.56)	19 (29.69)	0	0	50 (78.13)	30 (46.88)
5–9	29 (1.91)	5 (17.24)	4 (13.79)	5 (17.24)	4 (13.79)	5 (17.24)	8 (27.59)	15 (51.72)	16 (55.17)
10–17	36 (2.37)	19 (52.78)	10 (27.78)	12 (33.33)	7 (19.44)	8 (22.22)	7 (19.44)	39 (108.33)	24 (66.67)
18–30	175 (11.51)	87 (49.71)	52 (29.71)	50 (28.57)	28 (16.00)	34 (19.43)	47 (26.86)	171 (97.71)	127 (72.57)
30–65	627 (41.22)	234 (37.32)	138 (22.01)	320 (51.04)	176 (28.07)	126 (20.10)	161 (25.68)	680 (108.45)	475 (75.76)
≥65	590 (38.79)	219 (37.12)	130 (22.03)	215 (36.44)	124 (21.02)	55 (9.32)	71 (12.03)	489 (82.88)	325 (55.08)
Total	1521	581 (38.20)	345 (22.68)	635 (41.75)	358 (23.54)	228 (14.99)	294 (19.33)	1444 (94.94)	997 (65.55)
*p*-value		0.0779		0.0863		0.0812		0.0830	

HI: hemagglutination inhibition assay; ELISA: enzyme-linked immunosorbent assay. ^1^ Data represent the number of positive samples (percentage).

## Data Availability

The original contributions presented in this study are included in the article. Further inquiries can be directed to the corresponding author.

## Abbreviations

The following abbreviations are used in this manuscript:

rtRT-PCR	Real-time polymerase chain reaction
ARVI	Acute respiratory viral infection
ELISA	Enzyme-linked immunosorbent assay
ELISA TS IgG	Enzyme-linked immunosorbent assay system for the determination of antibody class IgG
HI	Hemagglutinating activity inhibition
IDS	Influenza diagnostic serum, dry
hAdv	Adenovirus
hBov	Bocavirus
hCov	Coronavirus
hMpv	Metapneumovirus
hPiv	Paramyxovirus
hRSv	Respiratory syncytial virus
hRv	Rhinovirus
